# Influence of High Temperature Curing and Surface Humidity on the Tensile Strength of UHPC

**DOI:** 10.3390/ma14154260

**Published:** 2021-07-30

**Authors:** Matthias Kalthoff, Michael Raupach, Thomas Matschei

**Affiliations:** Institute of Building Materials Research (IBAC), RWTH Aachen University, Schinkelstraße 3, 52062 Aachen, Germany; raupach@ibac.rwth-aachen.de (M.R.); matschei@ibac.rwth-aachen.de (T.M.)

**Keywords:** direct tensile strength, ultra-high-performance concrete (UHPC), autoclaving, heat treatment

## Abstract

The objective of this study is an investigation of the different parameters that influence the tensile strength of ultra-high performance concrete (UHPC). Apart from the shrinkage and stiffness, the tensile strength is an important parameter for the design of crack-free concrete elements, e.g., in machine tool construction. One focus of our work is the influence of concrete curing and the great impact of the mechanical and physical characteristics of hydrated UHPC. For this reason, different curing regimes were investigated. The results show that even after heat treatment or autoclaving, the centric tensile strength of UHPC specimens is strongly influenced by the surrounding ambient humidity. Test specimens that were stored under water after a heat treatment or autoclaving and were still wet during the test had the highest tensile strengths. Storage at 20 °C and 65% relative humidity (rH), however, results in a 25% reduction in tensile strength. Alternating storage between water storage at 20 °C water and storage at 65% rH can also reduce the tensile strength dramatically by up to 70%. In particular, samples that were stored at 65% rH right before testing had very low tensile strengths. Surprisingly, the initially low tensile strength of previously dry stored UHPC can be restored by subsequent water storage. In the absence of any microstructural defects, e.g., microcracks, a possible explanation for this phenomenon can be the stress differences due to a humidity gradient between the core and surfaces and shrinkage combined with a continued reaction of the unhydrated binders of the UHPC.

## 1. Introduction

The main objective of this work was the development of a suitable concrete composition that meets the high technical requirements of machine tool constructions [1,2]. During the use of machine tools, a drop in stiffness or even cracks in the construction has to be prevented. Furthermore, the material must not significantly deform as a result of environmental influences, e.g., humidity and temperature changes and the applied load [3] Currently, most machine tool constructions are still made out of steel because of its strength and stiffness. This is the reason why—unlike in the construction industry—the mechanical parameters, tensile strength and stiffness are typically used for the design of machine tools in mechanical engineering [4,5,6,7,8]. In addition to the application in machine tool construction, the tensile strength of concrete is also of great importance for the application and testing of textile concrete. In order to assess the load-bearing behaviour, reinforced and non-reinforced samples are tested in that respect and the tensile strength is determined. Since concrete used for textile concrete applications is usually of high to ultra-high strength, therefore, the results of this work are also of great importance to the textile concrete community. Moreover, the tensile strength of concrete is also important in case of fire since it has a significant influence on spalling and cracking due to high temperatures. For UHPC in particular, spalling behaviour can be a critical property because of the high elastic modulus and, thus, the extremely brittle cracking behaviour of UHPC [9,10,11].

A particular challenge is the correct testing of the tensile strength. At present, there is no standardised method for determining the centric tensile strength of concrete specimens. For this reason, the tensile strength is derived from auxiliary variables such as splitting strength and flexural tensile strength. The ratio of bending tensile strength to tensile strength corresponds to about 50% and for the splitting test approximately 90%. The fact that these methods are not transferable, especially for UHPC, was already shown in [12]. The choice of a suitable test method is essential for the reliable and reproducible determination of the concrete’s tensile strength. The tensile strength is influenced by several parameters. These mainly include the concrete composition, environmental conditions, test setup [13], specimen geometry [12], loading speed [14,15,16,17] or a possible preload.

Compared to the determination of the compressive strength, the measurement of the centric tensile strength is much more demanding due to the eccentricities that occur during the test and that additionally affect the specimens [18]. The literature describes numerous experiments for determining the centric tensile strength. As a general rule, the distinction is made between two test setups. In a first configuration the specimens are ground until the opposite surfaces are parallel to one another. Subsequently at these surfaces, adapter plates are adhesively attached and the tensile strength is tested after drying [19]. In a second method, the specimens are clamped at their ends during the tensile test [20].

Moreover, the concrete age and the selected curing method also affect the tensile strength [21,22]. The humidity can especially influence the strength of UHPC [23,24,25,26]. For example, the drying of the concrete surface, due to insufficient curing, can result in shrinkage cracks on the surface that induces a negative effect on the tensile strength. Furthermore, the service conditions have a great influence on the self-healing potential of the UHPC [27]. It is generally known that the compressive strength and tensile strength increase with the increasing age of the concrete [28,29]. Additionally, autoclaving or heat treatment can increase both early strength and final strength [19,30,31].

The results of [32] also showed that the flexural tensile strength tended to be negatively affected by post-treatment at temperatures >300 °C. This effect could be decreased slightly by the addition of microfibers, e.g., Kang et al. [33] conducted studies on the effect of post-treatment at 60 °C and 90 °C on the compressive and flexural tensile strength of UHPC. The results show that curing at 60 °C already results in a significant increase in compressive strength. The flexural tensile strength of the concrete was tested after 28 days. The test specimens were thereby stored after heat treatment for at least 23 days at 20 °C and 65% relative humidity before testing. The results show no clear effect of heat treatment on flexural tensile strength evolution. Wu et al. [34] performed three post-treatment regimes in their investigations. In those studies, the UHPC specimens were stored after demoulding (1) continuously under water at 20 °C, (2) under water at 90 °C for 48 h followed by storage at room temperature or (3) steam-cured at 90 °C for 48 h followed by storage at room temperature. The results by Wu et al. showed that post-treatment (2) had a positive effect on compressive and flexural strength compared to storage (1) under water at 20 °C. This is also attributed to an increase in rate of hydration of the binder. They concluded that the compressive strength is more influenced than the flexural strength.

As the centric tensile strength is a decisive parameter for the design of concrete structures, e.g., in machine tool construction, the results of an extensive investigation on the impact of varying curing and service conditions on the tensile strength of UHPC are presented below.

## 2. Materials and Methods

### 2.1. Materials

In this work, the influence of a heat treatment or autoclaving and the effect of the subsequent storage conditions on the tensile behaviour of UHPC were investigated. Two different UHPC compositions were investigated. The respective mix designs are shown in Table 1. UHPC 1 is a low carbon composition based on a CEM III/A which was also used in other studies [35] and UHPC 2 represents a traditional UHPC based on a CEM I 52.5 R. Bot mixes contained silica fume as supplementary cementitious material. Quartz powder and sand was used as aggregates with a maximum grain size of 0.5 mm. In order to investigate the influence of the different curing condition on the strength of the UHPC, test specimens were produced to determine the compressive strength and the tensile strength. One series consisted of three cubes each with a length of 100 mm and six tensile bone samples according to ASTM C307-03 [20] (heat treated series) and seven tensile bone samples (autoclaved series). The same geometry as in [13] was used for the tensile bones. The length of the specimen was 228.6 mm and the smallest cross-sectional area was 76.5 mm × 76.5 mm.

### 2.2. Methods

#### Curing Regimes

**Figure 1:**
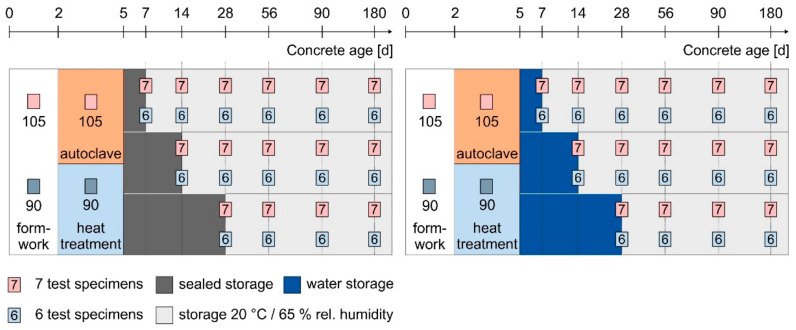
Overview of the curing variants, testing age and number of samples for water storage and sealed storage of UHPC 1.

Extensive experiments were carried out to investigate the influence of storage conditions after heat treatment or autoclaving on the tensile behaviour of UHPC 1. An overview of the variants of the curing conditions is provided in Figure 1. A total of 210 (2 × 105) specimens were autoclaved and 180 (2 × 90) specimens were heat treated.

Due to the use of slow hardening slag cement in UHPC 1 and the need of high contents of superplasticizer, all 390 test specimens made of UHPC were demoulded at the earliest 48 h after casting. Afterwards, heat treatment or autoclaving was applied. During heat treatment, the specimens were stored for 48 h in water at 90 °C. The heating rate from 20 °C to 90 °C was on average 7.0 °C/h and the cooling rate 4.0 °C/h.

During autoclaving, the specimens were stored in a laboratory autoclave. It was heated from 20 °C to approximately 190 °C and 12.5 bar saturation pressure within 9 h. The specimens remained there for another 15 h. Afterwards, the pressure was reduced to 6 bar and the heating system was switched off. Within 30 h, the specimens were removed from the autoclave again at 20 °C. Subsequently the specimens were stored for another 2, 9 or 23 days either under water at 20 °C or sealed storage by wrapping them into foil. At the end of the second curing step, the specimens were removed from the water or foil and stored at 20 °C and 65% relative humidity until testing.

**Figure 2:**
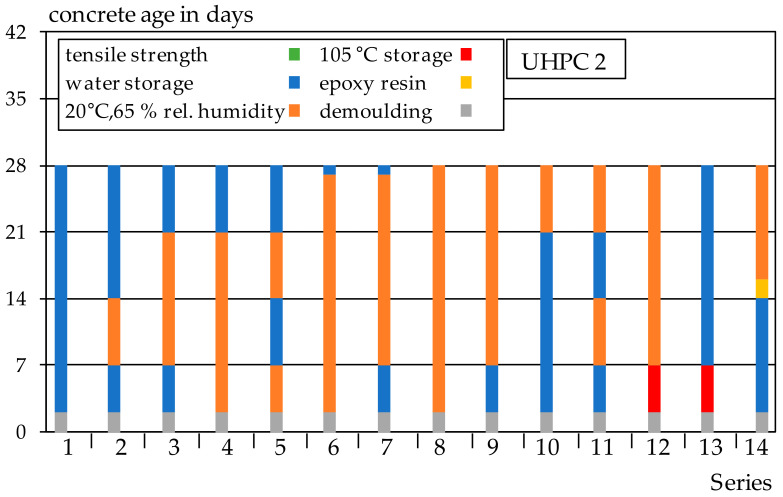
Overview of curing variants and their duration for UHPC 2.

Additionally, UHPC 2 was investigated without heat treatment and autoclaving. The test specimens remained in the formwork for 48 h and then exposed to different curing regimes. One series consists of 6 bone samples for the determination of the centric tensile strength and three cubes for the determination of the compressive strength. An overview of the different storage conditions can be observed in Figure 2. Water storage at 20 °C (blue), storage at 20 °C and 65% relative humidity (orange) and storage at 105 °C (red) were chosen as storage conditions. For Series 14, an additional layer of epoxy resin (yellow) was applied immediately after removal from the water. All specimens were tested at the age of 28 days and immediately after completion of the curing variants. Thus, for example, Series 3 was stored in the formwork for 48 h after manufacturing and then under water at 20 °C for 5 days. From a concrete age of 7 days, the specimens were stored for 14 days at 20 °C and 65% relative humidity and then again for 7 days under water at 20 °C.

### 2.3. Testing

**Figure 3:**
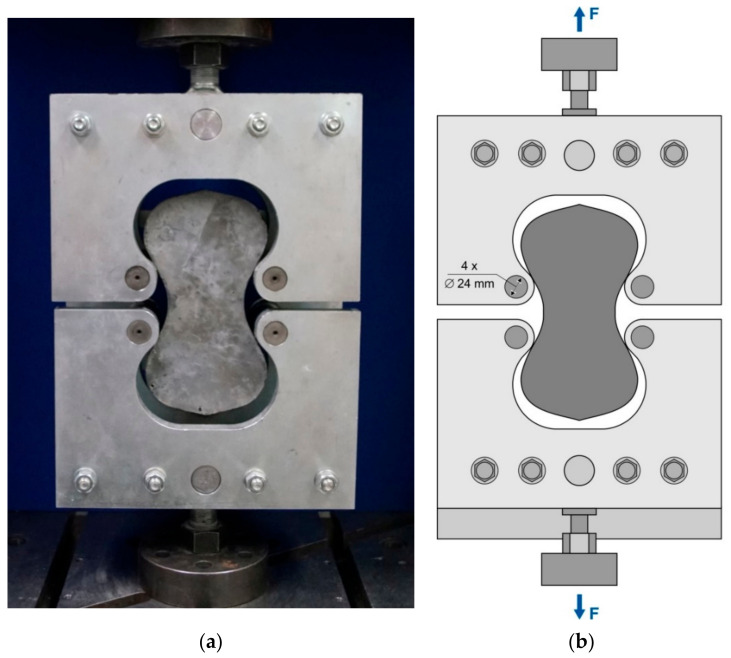
(**a**) The test setup for determining the centric tensile strength according to studies of Neunzig et al. [13]; (**b**) technical drawing of the test setup for determination of the centric tensile strength according to studies of Neunzig et al. [13].

In order to determine the centric tensile strength of UHPC 1 and UHPC 2 test specimens, the test set-up presented in [13] was applied. For this purpose, the “briquette test specimens” similar to ASTM C307-03 [19] were used with the geometric dimensions enlarged by a factor of 3. With this specimen size, it is possible to examine concretes with a maximum grain size of 16 mm. The geometry of the specimen has a cross-sectional area of approximately 76.5 mm × 76.5 mm at the smallest point depending on the filling height. The length of the specimen is 228.6 mm. The principal test setup is shown in Figure 3. The investigations from [13,19] show that the scattering is significantly lower compared to cylindrical test specimens. The four articulated rollers allow an almost centric force application into the specimen. Furthermore, complex specimen preparation such as plane-parallel grinding is no longer necessary. All test specimens were tested immediately after respective curing with a universal testing machine from Instron with a maximum force of 300 kN. The load was applied in a force-controlled manner so that the fracture occurred within 30–90 s in order to avoid a significant influence due to creep. In order to perform this, the first tensile bone sample was loaded until it failed. The loading speed was then adjusted and kept constant for the remaining specimens based on this result. This first specimen was not included in the evaluation. The test speeds were on average between 30 and 60 kN/min. The compressive strength was tested on additionally produced 100 mm cubes at a test speed of 5 kN/s according to the German standard DIN EN 12390-3 [36].

## 3. Results

### 3.1. Tensile Strength

#### 3.1.1. Heat Treatment

**Figure 4:**
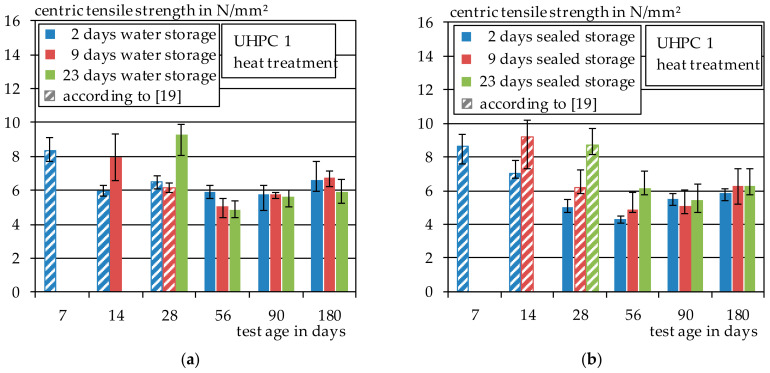
(**a**) Direct tensile strength of UHPC 1, heat treatment and water storage; (**b**) direct tensile strength of UHPC 1, heat treatment and sealed storage.

The results of the centric tensile tests for the heat-treated UHPC 1 test specimens are shown in Figure 4. In Figure 4a, the results of the tensile strength of the heat-treated test specimens with subsequent water storage of 2, 9 or 23 days are shown. Figure 4b contains the results the sealed storage of 2, 9 or 23 days. In addition, the results from earlier studies [19] are included as dashed lines.

The given scatter indicates the minimum and maximum test values for each series. The tensile strength of the UHPC 1 was determined at 7, 14, 28, 56, 90 and 180 days after manufacturing. The highest tensile strength values were reached by the test specimens that were stored under water before the testing and were also tested in wet conditions. This applies to the series that included 2 days of water/sealed storage, test age of 7 days, 9 days of water/sealed storage, test age of 14 days and the series that included 23 days of water/sealed storage and test age of 28 days. The tensile strength of the water/sealed samples varied between 8 N/mm^2^ and 10 N/mm^2^. The test specimens stored at 20 °C and 65% relative humidity—which applies to all other samples—after water or sealed storage showed a significant decrease in tensile strength. The average tensile strength of this series was only about 6 N/mm^2^. This is a 25% decrease in tensile strength compared to the water-stored specimens. At the age of 180 days, a slight increase in tensile strength can be observed again.

#### 3.1.2. Autoclave

**Figure 5:**
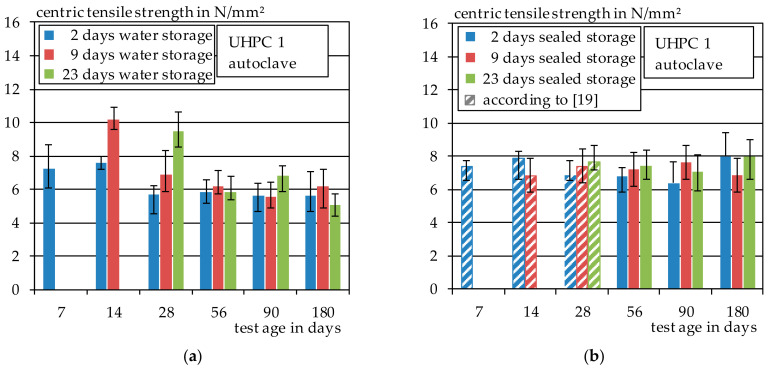
(**a**) Direct tensile strength of UHPC 1, autoclave and water storage; (**b**) direct tensile strength of UHPC 1, autoclave and seal-cured storage.

Similar results were also observed in case of the autoclaved series. The tensile strength of the autoclaved specimens with subsequent water storage of 2, 9 or 23 days are shown in Figure 5a and compared to the seal cured samples shown in Figure 5b. Again, water storage after autoclaving resulted in a significant increase in strength as long as the specimens were tested in wet conditions. In some cases, these even exceeded those of the heat-treated specimens. However, when the specimens are subsequently stored at 20 °C and 65% relative humidity, the tensile strength drops significantly again. Earlier results obtained in [19] are shown in dashed form.

No clear trend can be observed for the seal cured specimens. Their average tensile strength is around 7 N/mm^2^. The test specimens showed a dry surface when they were removed from the autoclave. Therefore, the subsequent sealed storage after autoclaving does not seem to have the same influence on the tensile strength in comparison to the heat-treated specimens. Thus, subsequent water storage seems to increase strength only as long as the surface of the specimens did not dry during the previous curing step. However, it is remarkable that water storage after autoclaving together with subsequent storage at 20 °C and 65% relative humidity seems to result in lower strengths compared to dry seal cured storage. The ambient humidity only seems to have a positive effect on the tensile strength as long as it is maintained.

**Figure 6:**
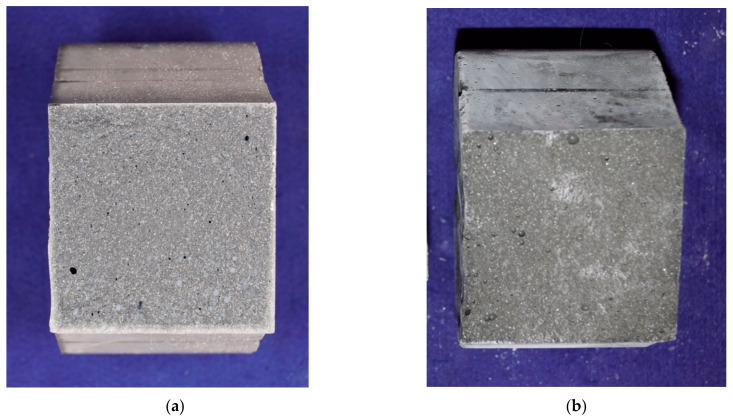
(**a**) Cross section of a UHPC specimen at 180 days of age after testing, autoclaved, 2 days water storage and placed in 20 °C/65% relative humidity until testing. (**b**) Cross section of a UHPC specimen at 28 days of age after testing, autoclaved and 23 days water storage.

Figure 6a shows a UHPC specimen that was autoclaved and then stored in water for 2 days, followed by a curing at 20 °C and 65% relative humidity until testing at the age of 180 days. The edge of the specimen is clearly brighter than the rest of the concrete cross section. This indicates that the edge of the specimen is drier than the core, which indicates the existence of a small moisture gradient within the specimen between the concrete core and the concrete surface. Figure 6b shows a UHPC specimen that was autoclaved and then stored in water until testing at the age of 28 days. Here, the edge of the specimen is as dark as the centre of the concrete cross-section. Therefore, the edge here does not seem to be drier than the concrete core. A careful analysis of the surface showed that no cracks or microcracks were visible on the concrete surface of either specimens.

#### 3.1.3. Drying–Wetting Curing

**Figure 7:**
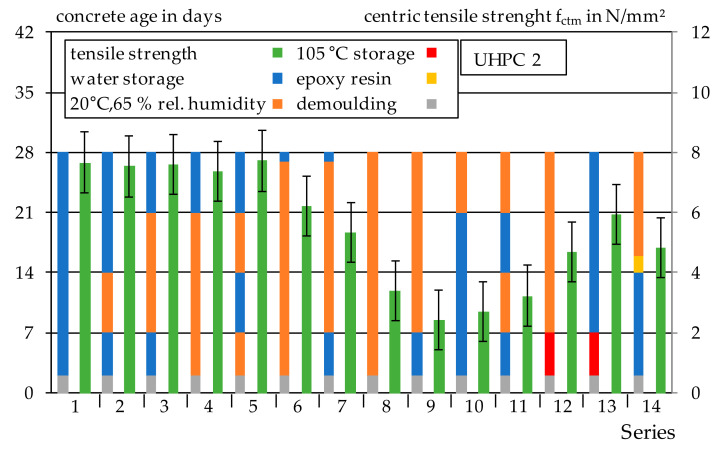
Results of the centric tensile strength of UHPC 2 and storage.

The results from Section 3.1.1 and Section 3.1.2 show that the tensile strength of UHPC is strongly dependent on the storage condition before the testing. In particular, moisture conditions seem to have a high influence. For this reason, no heat treatment or autoclaving was performed on UHPC 2. Instead, the specimens of the 14 series were exposed to different storage conditions until testing. The centric tensile strength and compressive strength were determined at the age of 28 days. The results of centric tensile tests and the corresponding storage are shown in Figure 7. The results show again that the highest tensile strengths are achieved if the specimens were stored under water for a sufficiently long period of time before the testing. The test specimens of Series 1–5, which were stored under water at 20 °C for a sufficiently long period of time before the test, show even higher tensile strengths of just under 8 N/mm^2^ than the test specimens stored at 20 °C and 65% relative humidity after heat treatment or autoclaving. Alternating storage between 20 °C and 65% relative humidity and water storage does result in a decrease in tensile strength as long as the specimens were stored under water again before the test. This is particularly obvious by comparing Series 5 and 11. Thus, a tensile strength can be restored by storage under water.

The comparison of Series 8 and 9 shows that water storage followed by storage at 20 °C and 65% relative humidity results in a reduction in tensile strength from 3.4 N/mm^2^ to 2.4 N/mm^2^. This corresponds to a loss of strength of about 30%. Similar effects could already be observed in Section 3.1.1 and Section 3.1.2. Storage of the test specimens at 105 °C for Series 12 and 13 results in an increase in tensile strength compared to L7 (Series 9) storage. However, the high strength values of Series 1–5 are not achieved with Series 12 and 13. Therefore, storage at 105 °C seems to have both a positive and negative effect on the tensile strength. In order to prevent drying effects, all specimens for determination of the tensile strength of Series 14 were coated with a special epoxy resin, which is especially suitable for use on moist concrete surfaces. The tensile strength of Series 14 is about 4.8 N/mm^2^ and is about 37% lower in comparison with Series 1, which is stored in water. The use of the epoxy resin has, thus, improved the tensile strength compared to the L7 storage (Series 9), however, the high tensile strength of Series 1–5 could not be achieved. This may indicate that the applied epoxy resin layer is not sufficiently diffusion resistant.

### 3.2. Compressive Strength

#### 3.2.1. Heat Treatment

**Figure 8:**
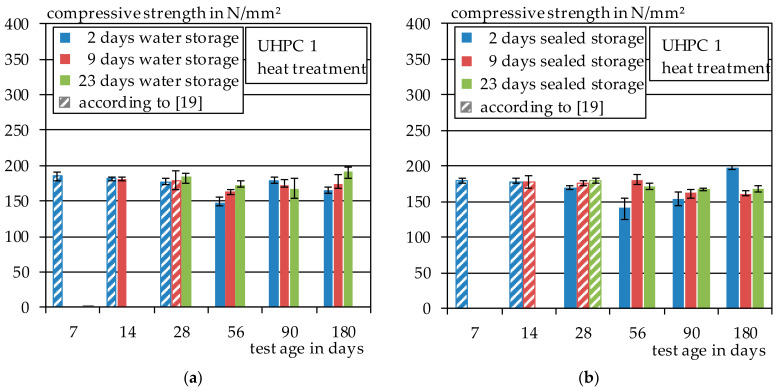
(**a**) Compressive strength of UHPC 1, heat treatment and water storage; (**b**) compressive strength of UHPC 1, heat treatment and sealed storage.

The results of the compressive tests for the heat-treated UHPC 1 test specimens are shown in and Figure 8a. In Figure 8b, the results of the compressive strength of the heat-treated test specimens with subsequent water storage of 2, 9 or 23 days are shown. In Figure 8b, the results of those in sealed storage of 2, 9 or 23 days are shown. Moreover, the results that have already been presented in [19] are included as dashed lines. No clear trend can be observed in the heat-treated specimens as a result of the different curing variants. With water storage, the average compressive strength of all test specimens is around 175 N/mm^2^ and with sealed storage of 171 N/mm^2^. Only at the age of 56 days is a decrease in compressive strength observed with the specimens that were stored for two days in foil and for two days under water. However, this seems to be more of an exception as the specimens show similar strengths again at the age of 90 and 180 days.

#### 3.2.2. Autoclave

**Figure 9:**
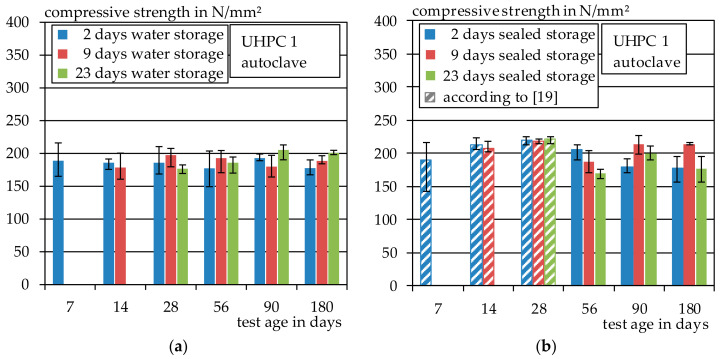
(**a**) Compressive strength of UHPC 1, autoclave and water storage; (**b**) compressive strength of UHPC 1, autoclave and sealed storage.

The results of the compressive strength test of the autoclaved specimens of UHPC 1 are shown in Figure 9. In comparison to the heat-treated specimens, the autoclaved specimens show significantly higher compressive strengths. The average compressive strength of all specimens that were stored under water is about 187 N/mm^2^ and, for the specimens that were stored in foil, about 200 N/mm^2^. However, the scattering within a series is higher compared to the heat-treated specimens from Section 3.2.1.

This is probably related to the increasing embrittlement of the specimens due to autoclaving. The results show that water storage after autoclaving with subsequent storage at 20 °C and 65% relative humidity tends to have a negative effect on the compressive strength. The same effect has already been observed with the tensile test specimens in Section 3.1.2. The results that have already been presented in [19] are shown as dashed lines.

#### 3.2.3. Drying–Wetting Curing

**Figure 10:**
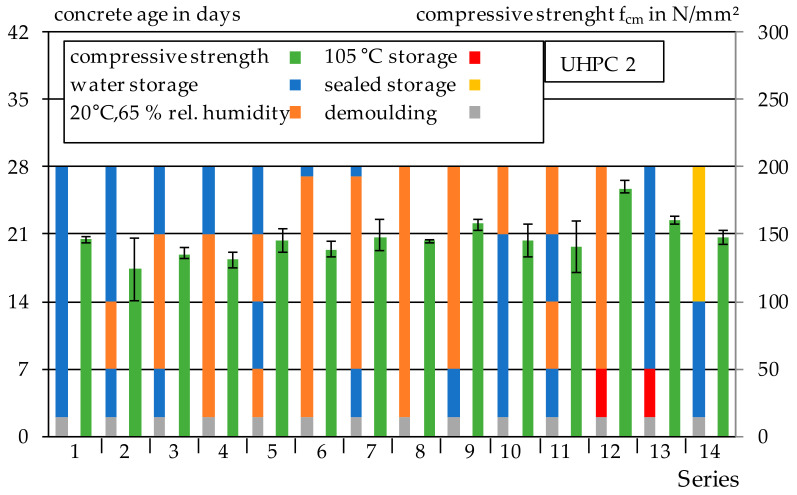
Results of the compression strength of UHPC 2 and storage.

The results of compressive tests and the corresponding curing conditions are shown in Figure 10. As expected, the compressive strengths are significantly lower compared to heat treatment and autoclaving. The average compressive strength of the Series 1–11 is 141 N/mm^2^ and is, thus, about 30% lower than that of the autoclaved test specimens with sealed storage. Storage at 105 °C results in a significant increase in the compressive strength of Series 12 with 184 N/mm^2^ compared to Series 1–11. This corresponds to an increase of 23%. The high temperatures results in the additional activation of silica fume. The same effect could also be observed, e.g., in [1]. The comparison of Series 12 and 13 also shows that subsequent water storage after initial heat curing at 105 °C results in a decrease in compressive strength. The cubes of Series 14 were not coated by epoxy resin after removal from the water, such as the tensile samples, since otherwise the force application surfaces would not be straight. Instead, the cubes were stored in foil after removal from water. All relevant data for this paper are presented in Appendix A, Table A1, Table A2, Table A3, Table A4, Table A5, Table A6, Table A7, Table A8, Table A9, Table A10, Table A11, Table A12, Table A13, Table A14, Table A15, Table A16, Table A17, Table A18, Table A19, Table A20, Table A21, Table A22, Table A23, Table A24, Table A25, Table A26, Table A27, Table A28 and Table A29.

## 4. Discussion

The results show that tensile strength is strongly influenced by moisture gradients even after heat treatment and the autoclaving. Three possible explanations will be discussed more in detail within the following working hypothesis.

### Working Hypothesis

The possible reasons for the different tensile strengths may be (i) the build-up of internal pre-stressing due to a gradient of humidity between the concrete core and the concrete surface, (ii) crack formation as a result of shrinkage during drying and (iii) the development of possible secondary reactions of the non-hydrated cement.

**Figure 11:**
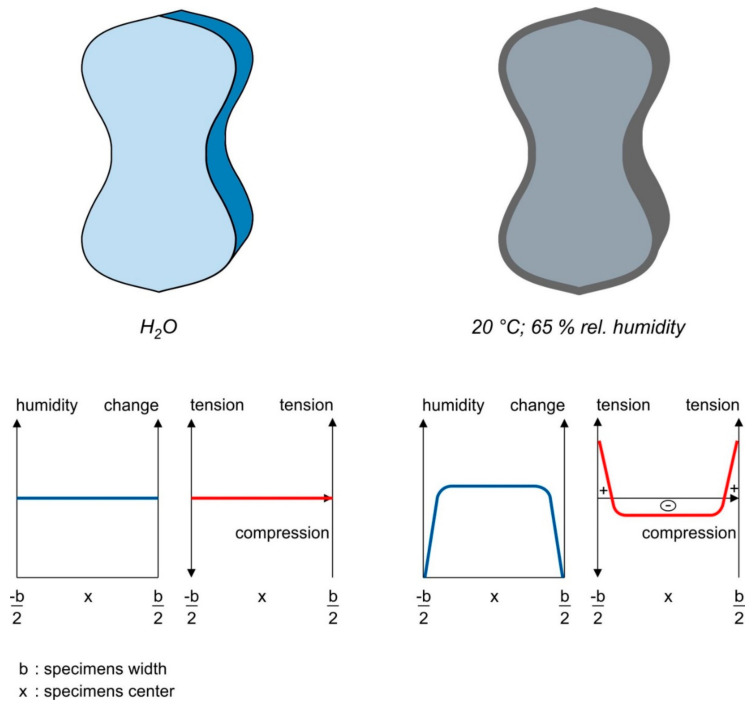
Residual stress due to different moisture levels.

The possible influence of the moisture gradient is shown in Figure 11. The specimen on the left is surrounded by water, which results in a constant moisture profile between the core and edge. Consequently, this means that there should be no additional residual stresses inside the wet specimens. In the right specimen, the surface of the concrete is ex-posed to 20 °C and 65% relative humidity. This causes the moisture at the edge of the concrete to decrease and creates a moisture gradient between the concrete core and concrete surface. This results in the occurrence of tensile stresses in the outer zone of the specimen. In the core of the sample, compressive stresses dominate, whereas, at the edge tensile, the stresses formed causes an internal prestressing of the outer zone. However, since there are no visible cracks, the occurring stress most likely does not exceed the tensile strength of the concrete.

When the test specimens are stored in water, it can be assumed that an inequality state between core and surface is established. If this state is changed, the different amount of water from the core to the surface causes a tensile prestressing within the specimen. This reduces the centric tensile strength of the specimens stored in dry conditions. As the specimens have already been subjected to heat treatment or autoclaving before the water storage, the influence of the hydration progression will not have a major influence on subsequent hardening as the development of strength has already been completed as a result of the high temperatures. This hypothesis also fits with the fracture figures Figure 6a,b where the dry edge was found on the test specimen stored at 20 °C and 65% relative humidity. The small increase in tensile strength after 180 days when stored at a temperature of 20 °C and 65% relative humidity indicates that the moisture state between the core and the surface is slowly equalizing and, thus, the prestress is relieved.

These results are also consistent with the results from [24], where the influence of moisture on creep was investigated. Bažant was also able to determine the influence of moisture distribution in normal concrete on the creep behaviour in the investigations. It is remarkable that, with UHPC, despite the dense microstructure and the almost non-existent capillary pores, moisture transport nevertheless occurs even though it is limited to the outer layer of a few millimeters. In addition, the mixing water is chemically and physically bound by the binder. This may result in self-desiccation effects. The reason why moisture transport nevertheless takes place is probably due to low capillary transport and diffusion effects. The exact cause cannot yet be determined at this point.

It is known from the literature [19] that cement-bound components swell or shrink depending on the ambient humidity. As a result of shrinkage, cracks may occur if the local tensile strength is exceeded. When the specimens are removed from the moist environment, drying shrinkage occurs in the specimens. This is not very distinctive for UHPC compared to normal concrete, since the autogenous shrinkage of UHPC exceeds the autogenous shrinkage of normal concrete. Nevertheless, the drying shrinkage can result in cracks on the surface of the test specimens. These cracks would act similar to a predetermined breaking point in the tensile test, which significantly reduces the tensile strength of the brittle UHPC. However, with Series 3–5 of the UHPC 2, the tensile strength could be almost fully restored by sufficiently long periods of water storage. Thus, the shrinkage cracks seem to heal again or the cracks do not seem to have a great influence on the tensile strength. This influence cannot be exactly estimated yet at this point. It can be assumed that self-healing processes would typically take much longer than the typical timeframe in which the strength recovery was observed.

The low water/binder content of the UHPC mixture used in this work has a value of 0.19 for UHPC 1 und 0.21 for UHPC 2. This means that only some part of the used cement is chemically and physically bounded. Thus, secondary reactions of the non-hydrated cement can take place during subsequent water storage. These can also have a positive influence on the tensile strength. The results of this work show that curing variants still influences the material properties, especially the tensile strength, even after heat treatment or autoclaving. The effects are sometimes significant and should be taken into account when designing components where the tensile strength of the concrete plays a decisive role, such as in machine tool construction. Since machine tools are usually cooled with water and thus exposed to changing humidity conditions, UHPC components should be autoclaved and coated with a diffusion-proof layer such as epoxy resin. In this manner, the reduction in tensile strength can be prevented by water.

This section may be divided by subheadings. It should provide a concise and precise description of the experimental results, their interpretation, as well as the experimental conclusions that can be drawn.

## 5. Conclusions

The tensile strength of UHPC is of decisive importance for some applications in machine tool construction or textile concrete. Due to the fact that these constructions must meet high technical requirements, a comprehensive test program was carried out. The focus of this work was to investigate the tensile strength of UHPC up to an age of 180 days. In addition, different types of curing were investigated. With the help of the investigations, the effect of the post-treatment on the tensile strength and the compressive strength could be described in detail and can be used for the dimensioning the components in machine tool construction. The main results of this paper are listed as follows:

- The tensile strength of UHPC can be increased by both autoclaving and heat treat-ment. In addition, it is shown that the tensile strength depends significantly on the environmental conditions after autoclaving and heat treatment. The highest tensile strengths were determined during subsequent water storage and reached 10 N/mm^2^. The curing of the test specimens at 20 °C and 65% relative humidity resulted in a de-crease in tensile strength to around 6 N/mm^2^. This applies to both water and sub-sequent sealed storage.
- No definite trend can be observed in the specimens that were autoclaved before sealed storage. The individual values scatter comparatively strongly and are around 7 N/mm^2^.
- Water storage seems to increase the tensile strength. The curing of the specimens at 20 °C and 65% relative humidity results in a reduction in tensile strength. This phenomenon can be explained by shrinkage during drying and the resulting cracks can be explained by the stress gradient between the moist concrete core and the dry surface. As the drying shrinkage of UHPC is significantly lower than its autogenous shrinkage and the possible hydration reactions at the given amount of water due to the heat treatment is far proceeded, it can be assumed that the influence of the humidity gradient between the core and surface is the main influence.
- The influence of possible self-healing or secondary reactions of the non-hydrogenated binder cannot be sufficiently assessed at this point.
- Coating the specimens in wet condition with epoxy resin resulted in higher tensile strengths compared to storage at 20 °C and 65% relative humidity and lower tensile strengths compared to water storage. Thus, a coating with epoxy resin does not seem to be able to sufficiently maintain the moisture content of the concrete surface, resulting in a reduction in tensile strength.
- The highest compressive strength was found in autoclaved specimens. In the case of autoclaved test specimens, subsequent water storage results in a reduction in compressive strength. The phenomenon could not be fixed during heat treatment.

## 6. Outlook

Based on the results, the influence of the pre-stressing due to water storage will be investigated in more detail. In addition, extensive shrinkage tests are carried out under changing storage conditions in order to investigate any shrinkage crack formation that may occur. Furthermore, the influence of fibres on the tensile strength in connection with curing and different moisture conditions will be investigated. The results of the study can be a great contribution in the design and manufacturing of concrete machine tools. The authors aim to apply the attained knowledge in practice to generate and further improve industry cooperation projects.

## Figures and Tables

**Table 1:** Mixture composition of UHPC.

Parameter	Unit	UHPC 1	UHPC 2
CEM III/A 52.5 R	kg/m^3^	832	-
CEM I 52.5 R		-	750
Water		189	189
quartz powder 0–0.063 mm		219	417
quartz powder 0–0.250 mm		-	245
River sand 0.1–0.5 mm		1.030	444
Silica fume		135	158
Superplasticizer	M.-% of cement	3.53	5.00
*w*/*b*-ratio ^(1)^	-	0.19	0.21

^(1)^ *w*/*b* value: water binder ratio.

## Data Availability

The relevant data are presented in Appendix A.

## Appendix A

**Table A1:** Tensile strength up to 180 days; UHPC 1; curing 1 heat treatment in 3 days; curing 2 water in 2 days.

Testing Age	No.	Single Value	Average	Standard Deviation	Coefficient of Variation	Error Indicator	
d		N/mm^2^			%	+	-
7 ^(1)^	1	8.4	8.3	0.53	6.43	0.77	0.61
	2	7.7					
	3	8.6					
	4	7.7					
	5	9.1					
14 ^(1)^	1	5.9	5.9	0.25	4.22	0.38	0.28
	2	6.3					
	3	5.7					
	4	5.7					
	5	6.1					
28 ^(1)^	1	6.1	6.5	0.35	5.36	0.40	0.42
	2	6.9					
	3	6.1					
	4	6.6					
	5	6.8					
56	1	6.3	5.9	0.31	5.28	0.3	0.4
	2	6.1					
	3	5.6					
	4	5.5					
	5	6.1					
90	1	6.1	5.7	0.54	9.39	0.56	0.94
	2	6.0					
	3	6.3					
	4	5.5					
	5	4.8					
180	1	5.9	6.6	0.68	10.30	1.03	0.73
	2	6.1					
	3	7.2					
	4	7.7					
	5	6.3					

^(1)^ According to [19].

**Table A2:** Tensile strength up to 180 days; UHPC 1; curing 1 heat treatment in 3 days; curing 2 water in 9 days.

Testing Age	No.	Single Value	Average	Standard Deviation	Coefficient of Variation	Error Indicator	
d		N/mm^2^			%	+	-
14	1	9.4	8.0	1.18	14.79	1.39	1.38
	2	6.6					
	3	6.9					
	4	9.4					
	5	7.7					
28 ^(1)^	1	6.2	6.2	0.20	3.30	0.24	0.28
	2	6.3					
	3	6.0					
	4	6.4					
	5	5.9					
56	1	5.5	5.1	0.43	8.53	0.4	0.66
	2	4.7					
	3	5.4					
	4	4.4					
	5	5.3					
90	1	5.8	5.8	0.15	2.67	0.10	0.30
	2	5.5					
	3	5.9					
	4	5.9					
	5	5.9					
180	1	6.3	6.7	0.32	4.73	0.39	0.47
	2	6.9					
	3	6.5					
	4	6.9					
	5	7.1					

^(1)^ According to [19].

**Table A3:** Tensile strength up to 180 days; UHPC 1; curing 1 heat treatment in 3 days; curing 2 water in 23 days.

Testing Age	No.	Single Value	Average	Standard Deviation	Coefficient of Variation	Error Indicator	
d		N/mm^2^			%	+	-
28	1	9.9	9.3	0.76	8.22	0.62	1.27
	2	9.9					
	3	9.9					
	4	8.8					
	5	8.0					
56	1	4.6	4.9	0.36	7.32	0.54	0.46
	2	4.8					
	3	4.4					
	4	5.4					
	5	5.1					
90	1	5.9	5.6	0.38	6.78	0.36	0.64
	2	5.0					
	3	6.0					
	4	5.9					
	5	5.4					
180	1	6.5	5.9	0.59	9.96	0.77	0.64
	2	5.8					
	3	6.7					
	4	5.3					
	5	5.2					

**Table A4:** Tensile strength up to 180 days; UHPC 1; curing 1 heat treatment in 3 days; curing 2 sealing in 2 days.

Testing Age	No.	Single Value	Average	Standard Deviation	Coefficient of Variation	Error Indicator	
d		N/mm^2^			%	+	-
7 ^(1)^	1	9.3	8.6	0.61	7.08	0.70	1.06
	2	8.4					
	3	7.6					
	4	9.1					
	5	8.8					
14 ^(1)^	1	6.7	7.0	0.38	5.47	0.73	0.32
	2	7.1					
	3	7.8					
	4	6.9					
	5	6.7					
28	1	5.5	5.0	0.28	5.66	0.50	0.30
	2	4.8					
	3	4.9					
	4	5.1					
	5	4.7					
56	1	4.5	4.3	0.17	3.89	0.20	0.20
	2	4.4					
	3	4.1					
	4	4.4					
	5	4.1					
90	1	5.8	5.5	0.27	4.88	0.30	0.40
	2	5.4					
	3	5.1					
	4	5.4					
	5	5.8					
180	1	5.4	5.8	0.24	4.08	0.27	0.42
	2	5.9					
	3	6.1					
	4	6.0					
	5	5.8					

^(1)^ According to [19].

**Table A5:** Tensile strength up to 180 days; UHPC 1; curing 1 heat treatment in 3 days; curing 2 sealing in 9 days.

Testing Age	No.	Single Value	Average	Standard Deviation	Coefficient of Variation	Error Indicator	
d		N/mm^2^			%	+	-
14 ^(1)^	1	9.6	9.2	0.96	10.38	0.62	1.91
	2	9.8					
	3	9.7					
	4	7.3					
	5	9.6					
28 ^(1)^	1	6.0	6.2	0.31	5.05	0.47	0.35
	2	5.9					
	3	6.7					
	4	6.0					
	5	6.5					
56	1	4.7	4.9	0.21	4.28	0.30	0.20
	2	5.2					
	3	5.1					
	4	4.8					
	5	4.7					
90	1	5.2	5.1	0.28	5.53	0.34	0.46
	2	5.2					
	3	4.9					
	4	5.4					
	5	4.6					
180	1	7.0	6.3	0.74	11.75	0.90	1.14
	2	5.9					
	3	5.2					
	4	6.3					
	5	7.2					

^(1)^ According to [19].

**Table A6:** Tensile strength up to 180 days; UHPC 1; curing 1 heat treatment in 3 days; curing 2 sealing in 23 days.

Testing Age	No.	Single Value	Average	Standard Deviation	Coefficient of Variation	Error Indicator	
d		N/mm^2^			%	+	-
28 ^(1)^	1	8.2	8.7	0.71	8.11	1.20	0.59
	2	8.3					
	3	9.9					
	4	9.1					
	5	8.1					
56	1	7.0	6.2	0.49	8.04	0.88	0.42
	2	5.7					
	3	6.4					
	4	5.8					
	5	5.8					
90	1	5.5	5.4	0.40	7.32	0.48	0.72
	2	5.4					
	3	4.7					
	4	5.9					
	5	5.6					
180	1	6.0	6.3	0.38	6.00	0.48	0.56
	2	6.4					
	3	6.5					
	4	6.8					
	5	5.7					

^(1)^ According to [19].

**Table A7:** Tensile strength up to 180 days; UHPC 1; curing autoclave in 3 days; curing 2 water in 2 days.

Testing Age	No.	Single Value	Average	Standard Deviation	Coefficient of Variation	Error Indicator	
d		N/mm^2^			%	+	-
7	1	6.9	7.2	0.80	10.90	1.50	1.10
	2	7.5					
	3	7.0					
	4	8.7					
	5	6.1					
	6	6.9					
14	1	7.5	7.6	0.30	4.40	0.40	0.40
	2	8.1					
	3	8.1					
	4	7.4					
	5	7.6					
	6	7.2					
28	1	6.2	5.7	0.58	10.14	0.54	1.15
	2	5.9					
	3	5.9					
	4	4.6					
	5	6.2					
	6	5.4					
56	1	5.2	5.8	0.50	8.00	0.80	0.60
	2	5.8					
	3	6.6					
	4	5.6					
	5	6.0					
	6	5.4					
90	1	6.0	5.6	0.58	10.27	0.78	0.91
	2	4.7					
	3	5.5					
	4	6.0					
	5	6.4					
	6	5.1					
180	1	5.0	5.6	0.80	14.15	1.43	0.99
	2	4.6					
	3	7.1					
	4	6.2					
	5	5.6					
	6	5.4					

**Table A8:** Tensile strength up to 180 days; UHPC 1; curing 1 autoclave in 3 days; curing 2 water in 9 days.

Testing Age	No.	Single Value	Average	Standard Deviation	Coefficient of Variation	Error Indicator	
d		N/mm^2^			%	+	-
14	1	10.9	10.2	0.50	4.70	0.70	0.60
	2	10.5					
	3	10.5					
	4	9.9					
	5	9.6					
	6	9.7					
28	1	7.2	6.9	0.80	11.70	1.40	1.00
	2	6.5					
	3	6.2					
	4	7.3					
	5	8.3					
	6	5.9					
56	1	6.5	6.2	0.50	7.70	0.90	0.50
	2	7.1					
	3	6.1					
	4	5.8					
	5	5.8					
	6	6.2					
90	1	6.4	5.6	0.51	9.08	0.83	0.67
	2	5.8					
	3	4.9					
	4	5.0					
	5	5.6					
	6	5.7					
180	1	6.7	6.2	0.80	13.60	1.00	1.30
	2	5.5					
	3	7.1					
	4	5.8					
	5	4.9					
	6	7.2					

**Table A9:** Tensile strength up to 180 days; UHPC 1; curing 1 autoclave in 3 days; curing 2 water in 23 days.

Testing Age	No.	Single Value	Average	Standard Deviation	Coefficient of Variation	Error Indicator	
d		N/mm^2^			%	+	-
28	1	9.9	9.5	0.70	7.30	1.10	1.00
	2	10.6					
	3	8.9					
	4	9.6					
	5	9.3					
	6	8.4					
56	1	5.8	5.8	0.50	7.80	1.00	0.40
	2	5.5					
	3	5.5					
	4	5.8					
	5	5.6					
	6	6.8					
90	1	6.6	6.8	0.50	7.30	0.60	0.90
	2	7.1					
	3	6.6					
	4	7.3					
	5	5.9					
	6	7.2					
180	1	5.4	5.1	0.43	8.59	0.71	0.66
	2	5.8					
	3	4.4					
	4	4.8					
	5	5.0					
	6	5.0					

**Table A10:** Tensile strength up to 180 days; UHPC 1; curing 1 autoclave in 3 days; curing 2 sealing in 2 days.

Testing Age	No.	Single Value	Average	Standard Deviation	Coefficient of Variation	Error Indicator	
d		N/mm^2^			%	+	-
7 ^(1)^	1	7.5	7.4	0.44	5.92	0.34	0.86
	2	7.6					
	3	7.6					
	4	6.5					
	5	7.7					
	6	7.4					
14 ^(1)^	1	8.2	7.9	0.60	7.61	0.43	1.23
	2	8.3					
	3	7.6					
	4	6.6					
	5	8.3					
	6	8.3					
28 ^(1)^	1	6.6	6.9	0.46	6.69	0.89	0.36
	2	6.6					
	3	6.9					
	4	7.8					
	5	6.5					
	6	6.9					
56	1	6.6	6.8	0.50	7.32	0.57	0.93
	2	7.1					
	3	6.6					
	4	7.3					
	5	5.9					
	6	7.2					
90	1	6.6	6.4	0.80	11.80	1.30	0.90
	2	5.5					
	3	7.7					
	4	5.5					
	5	6.5					
	6	6.6					
180	1	9.3	8.0	1.06	13.21	1.44	1.35
	2	7.0					
	3	9.5					
	4	7.6					
	5	6.7					
	6	8.1					

^(1)^ According to [19].

**Table A11:** T ensile strength up to 180 days; UHPC 1; curing 1 autoclave in 3 days; curing 2 sealing in 9 days.

Testing Age	No.	Single Value	Average	Standard Deviation	Coefficient of Variation	Error Indicator	
d		N/mm^2^			%	+	-
14 ^(1)^	1	6.9	6.9	0.16	2.30	0.14	0.29
	2	6.6					
	3	7.0					
	4	7.0					
	5	6.8					
	6	6.9					
28 ^(1)^	1	7.6	7.4	0.34	4.59	0.45	0.57
	2	7.5					
	3	7.3					
	4	6.8					
	5	7.9					
	6	7.4					
56	1	7.8	7.2	0.40	6.00	0.70	0.60
	2	7.6					
	3	7.1					
	4	6.9					
	5	6.6					
	6	6.9					
90	1	6.8	7.6	0.46	5.99	0.48	0.89
	2	7.6					
	3	8.1					
	4	7.8					
	5	8.1					
	6	7.5					
180	1	6.4	6.9	0.72	10.58	1.03	1.22
	2	5.6					
	3	7.0					
	4	7.4					
	5	6.8					
	6	7.9					

^(1)^ According to [19].

**Table A12:** Tensile strength up to 180 days; UHPC 1; curing 1 autoclave in 3 days; curing 2 and sealing 23 days.

Testing Age	No.	Single Value	Average	Standard Deviation	Coefficient of Variation	Error Indicator	
d		N/mm^2^			%	+	-
28 ^(1)^	1	7.5	7.7	0.38	4.90	0.62	0.51
	2	7.8					
	3	7.6					
	4	8.3					
	5	7.2					
	6	7.7					
56	1	6.6	7.4	0.70	9.90	1.40	0.80
	2	8.7					
	3	7.8					
	4	6.9					
	5	7.4					
	6	6.9					
90	1	7.3	7.1	0.78	10.98	0.78	1.17
	2	5.9					
	3	7.6					
	4	7.9					
	5	7.8					
	6	6.1					
180	1	9.3	8.0	1.10	13.20	1.40	1.40
	2	7.0					
	3	9.5					
	4	7.6					
	5	6.7					
	6	8.1					

^(1)^ According to [19].

**Table A13:** Compressive strength up to 180 days; UHPC 1; curing 1 heat treatment in 3 days; curing 2 water in 2 days.

Testing Age	No.	Single Value	Average	Standard Deviation	Coefficient of Variation	Error Indicator	
d		N/mm^2^			%	+	-
7 ^(1)^	1	191.0	186.2	5.7	3.1	4.9	8.0
	2	189.3					
	3	178.2					
14 ^(1)^	1	181.0	181.4	1.9	1.1	2.5	2.1
	2	179.3					
	3	183.9					
28 ^(1)^	1	182.1	177.7	3.8	2.1	4.5	4.8
	2	172.8					
	3	178.0					
56	1	155.2	147.3	5.6	3.8	7.9	4.7
	2	144.1					
	3	142.6					
90	1	184.5	179.1	3.8	2.1	5.4	3.2
	2	176.9					
	3	175.8					
180	1	170.4	165.4	3.8	2.3	5.0	4.1
	2	164.4					
	3	161.3					

^(1)^ According to [19].

**Table A14:** Compressive strength up to 180 days; UHPC 1; curing 1 heat treatment in 3 days; curing 2 water in 9 days.

Testing Age	No.	Single Value	Average	Standard Deviation	Coefficient of Variation	Error Indicator	
d		N/mm^2^			%	+	-
14	1	178.8	180.8	2.3	1.3	3.3	2.0
	2	179.5					
	3	184.1					
28 ^(1)^	1	192.5	179.0	10.6	5.9	13.4	12.4
	2	166.6					
	3	178.1					
56	1	164.7	163.9	3.0	1.8	3.1	4.0
	2	167.0					
	3	159.9					
90	1	179.4	172.9	4.7	2.7	6.6	3.9
	2	169.0					
	3	170.2					
180	1	186.6	175.0	8.2	4.7	11.6	6.8
	2	168.2					
	3	170.2					

^(1)^ According to [19].

**Table A15:** Compressive strength up to 180 days; UHPC 1; curing 1 heat treatment in 3 days; curing 2 water in 23 days.

Testing Age	No.	Single Value	Average	Standard Deviation	Coefficient of Variation	Error Indicator	
d		N/mm^2^			%	+	-
28	1	175.7	184.1	6.0	3.3	5.3	8.4
	2	187.1					
	3	189.4					
56	1	169.4	173.1	3.5	2.0	4.7	3.7
	2	177.8					
	3	172.0					
90	1	167.5	167.5	11.7	7.0	14.3	14.3
	2	181.7					
	3	153.1					
180	1	195.9	192.4	7.1	3.7	6.4	9.9
	2	182.4					
	3	198.8					

**Table A16:** Compressive strength up to 180 days; UHPC 1; curing 1 heat treatment in 3 days; curing 2 and sealing in 2 days.

Testing Age	No.	Single Value	Average	Standard Deviation	Coefficient of Variation	Error Indicator	
d		N/mm^2^			%	+	-
7 ^(1)^	1	182.8	179.7	3.0	1.7	3.1	4.0
	2	175.7					
	3	180.7					
14 ^(1)^	1	182.7	178.2	3.2	1.8	4.5	2.6
	2	175.6					
	3	176.3					
28	1	172.7	169.9	2.6	1.5	2.8	3.4
	2	166.5					
	3	170.4					
56	1	146.8	141.9	12.6	8.9	12.4	17.3
	2	154.3					
	3	124.6					
90	1	155.3	154.6	7.7	5.0	9.1	9.8
	2	163.6					
	3	144.8					
180	1	195.1	198.6	2.5	1.2	1.8	3.5
	2	200.4					
	3	200.4					

^(1)^ According to [19].

**Table A17:** Compressive strength up to 180 days; UHPC 1; curing 1 heat treatment in 3 days; curing 2 and sealing in 9 days.

Testing Age	No.	Single Value	Average	Standard Deviation	Coefficient of Variation	Error Indicator	
d		N/mm^2^			%	+	-
14 ^(1)^	1	169.1	177.7	7.3	4.1	9.1	8.6
	2	186.8					
	3	177.2					
28 ^(1)^	1	177.2	176.6	2.5	1.4	2.7	3.4
	2	173.2					
	3	179.3					
56	1	174.8	179.7	6.6	3.7	9.3	4.9
	2	175.3					
	3	189.0					
90	1	167.7	162.7	5.7	3.5	4.9	8.1
	2	165.9					
	3	154.7					
180	1	165.7	161.5	3.1	1.9	4.2	3.0
	2	158.5					
	3	160.3					

^(1)^ According to [19].

**Table A18:** Compressive strength up to 180 days; UHPC 1; curing 1 heat treatment in 3 days; curing 2 and sealing in 23 days.

Testing Age	No.	Single Value	Average	Standard Deviation	Coefficient of Variation	Error Indicator	
d		N/mm^2^			%	+	-
28 ^(1)^	1	182.5	178.6	3.0	1.7	3.9	3.4
	2	178.1					
	3	175.2					
56	1	175.6	172.4	3.5	2.0	3.2	4.9
	2	174.0					
	3	167.5					
90	1	169.4	167.3	1.6	1.0	2.1	1.8
	2	167.0					
	3	165.5					
180	1	163.9	168.6	3.4	2.0	3.3	4.7
	2	169.9					
	3	171.9					

^(1)^ According to [19].

**Table A19:** Compressive strength up to 180 days; UHPC 1, curing 1 autoclave in 3 days; curing 2 water in 2 days.

Testing Age	No.	Single Value	Average	Standard Deviation	Coefficient of Variation	Error Indicator	
d		N/mm^2^			%	+	-
7	1	186.0	189.1	20.80	11.00	27.00	23.80
	2	165.3					
	3	216.1					
14	1	175.7	186.0	7.34	3.95	5.59	10.37
	2	190.8					
	3	191.6					
28	1	178.5	185.9	17.89	9.62	24.66	17.21
	2	168.7					
	3	210.6					
56	1	203.8	177.5	22.30	12.50	26.30	28.10
	2	179.3					
	3	149.4					
90	1	199.1	192.9	4.43	2.30	6.20	3.90
	2	189.0					
	3	190.6					
180	1	175.8	177.8	9.40	5.30	12.40	10.40
	2	190.2					
	3	167.4					

**Table A20:** Compressive strength up to 180 days; UHPC 1; curing 1 autoclave in 3 days; curing 2 water in 9 days.

Testing Age	No.	Single Value	Average	Standard Deviation	Coefficient of Variation	Error Indicator	
d		N/mm^2^			%	+	-
14	1	160.7	178.7	16.50	9.30	21.90	18.00
	2	200.6					
	3	174.7					
28	1	207.9	197.5	12.70	6.40	10.30	17.90
	2	205.1					
	3	179.6					
56	1	204.6	192.9	15.70	8.20	11.60	22.20
	2	170.7					
	3	203.5					
90	1	178.5	179.9	13.51	7.51	17.20	15.80
	2	164.1					
	3	197.1					
180	1	184.1	188.7	5.70	3.00	8.00	4.60
	2	196.8					
	3	185.4					

**Table A21:** Compressive strength up to 180 days; UHPC 1; curing 1 autoclave in 3 days; curing 2 water in 23 days.

Testing Age	No.	Single Value	Average	Standard Deviation	Coefficient of Variation	Error Indicator	
d		N/mm^2^			%	+	-
28	1	169.6	176.9	5.40	3.00	5.50	7.30
	2	182.4					
	3	178.7					
56	1	194.6	186.0	11.20	6.02	8.55	15.82
	2	193.3					
	3	170.2					
90	1	213.1	205.4	10.60	5.20	7.70	15.00
	2	212.6					
	3	190.4					
180	1	198.2	201.0	2.76	1.37	3.77	2.77
	2	204.8					
	3	200.0					

**Table A22:** Compressive strength up to 180 days; UHPC 1; curing 1 autoclave in 3 days; curing 2 and sealing in 2 days.

Testing Age	No.	Single Value	Average	Standard Deviation	Coefficient of Variation	Error Indicator	
d		N/mm^2^			%	+	-
7 ^(1)^	1	143.0	190.6	33.73	17.70	26.45	47.60
	2	217.1					
	3	211.8					
14 ^(1)^	1	211.1	213.5	7.41	3.47	10.03	7.63
	2	205.9					
	3	223.5					
28 ^(1)^	1	225.0	220.2	4.82	2.19	4.87	6.56
	2	221.8					
	3	213.6					
56	1	213.1	205.4	10.59	5.16	7.72	14.97
	2	212.6					
	3	190.4					
90	1	191.3	179.0	9.02	5.04	12.33	8.97
	2	170.0					
	3	175.6					
180	1	156.7	177.4	15.77	8.89	17.48	20.73
	2	180.7					
	3	194.9					

^(1)^ According to [19].

**Table A23:** Compressive strength up to 180 days; UHPC 1; curing 1 autoclave in 3 days; curing 2 and sealing in 9 days.

Testing Age	No.	Single Value	Average	Standard Deviation	Coefficient of Variation	Error Indicator	
d		N/mm^2^			%	+	-
14 ^(1)^	1	217.2	208.0	6.60	3.17	9.21	5.88
	2	202.1					
	3	204.7					
28 ^(1)^	1	222.4	218.9	2.91	1.33	3.51	3.61
	2	215.3					
	3	219.0					
56	1	185.9	186.9	13.20	7.00	16.60	15.60
	2	203.5					
	3	171.3					
90	1	226.2	214.1	11.19	5.23	12.17	14.84
	2	199.2					
	3	216.7					
180	1	212.7	214.3	1.39	0.65	1.77	1.63
	2	216.1					
	3	214.2					

^(1)^ According to [19].

**Table A24:** Compressive strength up to 180 days; UHPC 1; curing 1 autoclave in 3 days; curing 2 and sealing in 23 days.

Testing Age	No.	Single Value	Average	Standard Deviation	Coefficient of Variation	Error Indicator	
d		N/mm^2^			%	+	-
28 ^(1)^	1	215.3	221.0	4.07	1.84	3.42	5.71
	2	223.3					
	3	224.4					
56	1	162.6	170.7	5.80	3.40	5.60	8.10
	2	176.3					
	3	173.2					
90	1	190.6	199.0	8.53	4.29	11.70	8.40
	2	195.7					
	3	210.7					
180	1	156.7	177.4	15.80	8.90	17.50	20.70
	2	180.7					
	3	194.9					

^(1)^ According to [19].

**Table A25:** Tensile strength; 28 days; UHPC 2 at concrete age of 28 days; Series 1–6.

Series	No.	Single Value	Average	Standard Deviation	Coefficient of Variation	Error Indicator	
		N/mm^2^			%	+	-
1	1	7.6	7.7	0.35	4.57	0.43	0.57
	2	8.1					
	3	7.1					
	4	7.5					
	5	7.9					
	6	7.8					
2	1	7.5	7.5	0.44	5.86	0.47	0.73
	2	7.9					
	3	7.3					
	4	6.8					
	5	7.7					
	6	8.0					
3	1	7.9	7.6	0.20	2.63	0.30	0.30
	2	7.5					
	3	7.6					
	4	7.6					
	5	7.7					
	6	7.3					
4	1	7.4	7.4	0.47	6.38	0.72	0.68
	2	7.4					
	3	7.6					
	4	8.1					
	5	6.7					
	6	7.1					
5	1	7.5	7.7	0.38	4.88	0.48	0.52
	2	7.5					
	3	7.2					
	4	8.2					
	5	8.0					
	6	7.9					
6	1	7.0	6.2	0.53	8.53	0.77	0.53
	2	6.0					
	3	5.9					
	4	6.8					
	5	5.7					
	6	6.0					

**Table A26:** Tensile strength; 28 days; UHPC 2 at concrete age of 28 days; Series 7–12.

Series	No.	Single Value	Average	Standard Deviation	Coefficient of Variation	Error Indicator	
		N/mm^2^			%	+	-
7	1	6.0	5.4	1.19	22.33	1.65	1.45
	2	3.9					
	3	7.0					
	4	5.2					
	5	4.1					
	6	5.9					
8	1	3.7	3.4	0.44	13.14	0.52	0.58
	2	3.9					
	3	3.6					
	4	3.4					
	5	2.8					
	6	2.9					
9	1	2.4	2.4	0.21	8.76	0.30	0.27
	2	2.7					
	3	2.2					
	4	2.6					
	5	2.1					
	6	2.4					
10	1	2.8	2.7	0.26	9.72	0.38	0.32
	2	2.9					
	3	2.5					
	4	2.4					
	5	2.6					
	6	3.1					
11	1	3.2	3.2	0.37	11.35	0.37	0.53
	2	3.5					
	3	3.5					
	4	2.9					
	5	3.6					
	6	2.7					
12	1	4.5	4.7	0.51	10.77	0.70	0.70
	2	5.1					
	3	4.0					
	4	4.8					
	5	5.4					
	6	4.4					

**Table A27:** Tensile strength; 28 days; UHPC 2 at concrete age of 28 days; Series 13–14.

Series	No.	Single Value	Average	Standard Deviation	Coefficient of Variation	Error Indicator	
		N/mm^2^			%	+	-
13	1	6.0	5.9	0.12	2.02	0.16	0.14
	2	5.8					
	3	5.8					
	4	6.1					
	5	5.9					
	6	6.0					
14	1	4.6	4.8	0.16	3.36	0.16	0.24
	2	4.7					
	3	4.8					
	4	5.0					
	5	4.9					
	6	5.0					

**Table A28:** Compressive strength; 28 days; UHPC 2 at concrete age of 28 days; Series 1–12.

Series	No.	Single Value	Average	Standard Deviation	Coefficient of Variation	Error Indicator	
		N/mm^2^			%	+	-
1	1	155.6	145.9	9.2	6.3	9.7	8.6
	2	137.3					
	3	144.9					
2	1	100.8	125.0	23.2	18.5	22.0	24.2
	2	147.0					
	3	127.1					
3	1	133.9	135.4	3.8	2.8	4.3	2.8
	2	132.6					
	3	139.7					
4	1	133.8	131.9	6.0	4.6	4.8	6.8
	2	125.1					
	3	136.7					
5	1	155.6	145.9	9.2	6.3	9.7	8.6
	2	137.3					
	3	144.9					
6	1	144.2	138.8	5.5	3.9	5.4	5.5
	2	133.3					
	3	138.8					
7	1	160.9	148.2	11.9	8.1	12.7	11.0
	2	146.5					
	3	137.2					
8	1	146.5	145.2	1.1	0.8	1.3	0.9
	2	144.9					
	3	144.3					
9	1	154.5	157.8	4.0	2.5	4.4	3.3
	2	162.2					
	3	156.7					
10	1	157.6	145.2	12.2	8.4	12.4	12.0
	2	144.7					
	3	133.2					
11	1	139.9	140.7	19.3	13.7	19.6	18.9
	2	121.8					
	3	160.3					
12	1	181.3	184.2	4.6	2.5	5.3	2.9
	2	189.5					
	3	181.8					

**Table A29:** Compressive strength; 28 days; UHPC 2 at concrete age of 28 days; Series 13–14.

Series	No.	Single Value	Average	Standard Deviation	Coefficient of Variation	Error Indicator	
		N/mm^2^			%	+	-
1	1	163.5	160.7	2.6	1.6	2.8	2.3
	2	160.3					
	3	158.4					
2	1	148.3	148.0	5.2	3.5	5.0	5.3
	2	153.0					
	3	142.7					
